# Syphilis and the Nervous System

**Published:** 1893-06

**Authors:** 


SYPHILIS AND THE NERVOUS SYSTEM; BEING A REVISED REPRINT OF THE LETTSONIAN LECTURES FOR 1890, DELIVERED BEFORE THE MEDICAL SOCIETY OF LONDON, by W. R. Gowers, M. D., F. R. C. P., J. R. S., Consulting Physician to University Hospital, Physician to the National Hospital for the Paralyzed and Epileptic, etc. Published by P. Blakiston, Son & Co., 1012 Walnut St., Philadelphia, 1892.

This publication is composed of three lectures delivered by the author, 1890, and with the additions mentioned in the preface, is presented to the profession as nearly complete as the times and original investigation will permit. We feel sure that much benefit will be gained by any one who may read it. To attempt to specialize the best points in these lectures were idle, for they are throughout, gems of the first water.

The author exhibits his usual force of expression, and at once impresses the reader with the truth of his observations and the depth of his research.

We make bold to state, that his pathology will stand for years, and is certainly a masterly description.

No better work is to be had on the subject, and it is to be hoped, the author will continue his investigation, for so important a subject, will, and does demand the time of him who is so well qualified to investigate. H. H.

